# T ^null ^and M ^null ^genotypes of the glutathione S-transferase gene are risk factor for CAD independent of smoking

**DOI:** 10.1186/1471-2350-7-38

**Published:** 2006-04-19

**Authors:** Khaled K Abu-Amero, Olayan M Al-Boudari, Gamal H Mohamed, Nduna Dzimiri

**Affiliations:** 1 Department of Genetics, King Faisal Specialist Hospital and Research Centre (MBC - 03), P. O. Box 3354, Riyadh 11211, Saudi Arabia; 2 Department of Biostatics, Epidemiology and Scientific Computing, King Faisal Specialist Hospital and Research Centre (MBC - 03), P. O. Box 3354, Riyadh 11211, Saudi Arabia

## Abstract

**Background:**

The association of the deletion in GSTT1 and GSTM1 genes with coronary artery disease (CAD) among smokers is controversial. In addition, no such investigation has previously been conducted among Arabs.

**Methods:**

We genotyped 1054 CAD patients and 762 controls for GSTT1 and GSTM1 deletion by multiplex polymerase chain reaction. Both CAD and controls were Saudi Arabs.

**Results:**

In the control group (n = 762), 82.3% had the T ^wild ^M ^wild^genotype, 9% had the T^wild ^M ^null^, 2.4% had the T^null ^M ^wild ^and 6.3% had the T^null ^M ^null ^genotype. Among the CAD group (n = 1054), 29.5% had the T^wild ^M ^wild ^genotype, 26.6% (p < .001) had the T^wild ^M ^null^, 8.3% (p < .001) had the T^null ^M ^wild ^and 35.6% (p < .001) had the T^null ^M ^null ^genotype, indicating a significant association of the T^wild ^M ^null^, T^null ^M ^wild ^and T^null ^M ^null ^genotypes with CAD. Univariate analysis also showed that smoking, age, hypercholesterolemia and hypertriglyceridemia, diabetes mellitus, family history of CAD, hypertension and obesity are all associated with CAD, whereas gender and myocardial infarction are not. Binary logistic regression for smoking and genotypes indicated that only M ^null ^and T^null^are interacting with smoking. However, further subgroup analysis stratifying the data by smoking status suggested that genotype-smoking interactions have no effect on the development of CAD.

**Conclusion:**

GSTT1 and GSTM1 null-genotypes are risk factor for CAD independent of genotype-smoking interaction.

## Background

Glutathione S-transferases (GSTs) are a group of enzymes known to play an important role in the detoxification of several endogenous and exogenous toxic and carcinogenic substances [1]. In humans, GST enzymes are divided into five subclasses: alpha (A), mu (M), pi (P), theta (T) and zeta (z). In addition, each class includes several genes and isoenzymes. GSTM1 products catalyze the conjugation of glutathione to epoxide derivatives of polycyclic aromatic hydrocarbons, the main carcinogens found in tobacco smoke. These products are important in the detoxification of naturally occurring monohalomethanes, dichloromethanes and ethylene oxides [1]. Polymorphisms in the GSTT1 and GSTM1 genes are caused by a deletion, which consequently results in virtual absence of enzyme activity, especially in individuals with deletion in both genes (null genotype). Many studies have demonstrated great concordance (> 95%) between the genotype and phenotype [2,3]. In experimental animals, chemicals in tobacco smoke have been reported to induce and stimulate atherosclerotic plaque formation [4]. Thus, deletions in the GSTT1 and GSTM1 genes affect the enzyme activity possibly contributing to the development of coronary atherosclerosis, especially among smokers [5-12]. The association of the GSTT1 and GSTM1 null-genotypes with coronary artery disease (CAD) among smokers has been the subject of many investigations [5-12], but to our knowledge no such studies have been conducted among Arabs. Since 3 decades ago, the incidence of CAD has been on the rise in Saudi Arabia. According to the biggest study conducted in this regard so far in the kingdom, the overall prevalence of CAD in Saudi Arabia is 5.5% [13]. This rise has been attributed to the major changes in the life-style of the Saudi population. High-fat diets, obesity, diabetes, and smoking, all of which are considered CAD risk factors, have become more prevalent, and people are leading a more sedentary lifestyle [14]. Therefore, our aim is to investigate the possible relevance of the GSTM1 and GSTT1 polymorphisms either individually or combined (haplotype) on the development of CAD. The study will also attempt to investigate the genotype-smoking interactions and their potential effect on the development of CAD in the presence or absence of other CAD risk factors.

## Methods

### Study population

The study group comprised 1054 individuals (642 males with a mean age of 52 ± 7.5 yr and 412 females with a mean age of 57 ± 7 yr) of Saudi Arabian descent with documented CAD, see Table 1 for demographic data on CAD patients. The inclusion criteria comprised, among others, the presence of angiographically determined narrowing of the coronary vessels by at least 70%, and can be defined as having severe disease. Exclusion criteria for CAD were coronary artery bypass surgery or angioplasty within 3months of the study, major cardiac rhythm disturbances, incapacitating or life-threatening illness, major psychiatric illness or substance abuse, history of cerebral vascular disease, neurological disorder, and administration of psychotropic medication. Additionally, 762 individuals (464 males, mean age 53 ± 6.1 yr and 298 females, mean age 55 ± 3.2 yr) undergoing surgery for heart valve diseases or receiving workups for cardiomyopathies and persons who reported with chest pain, but were established to have clear vessels by angiography, were recruited as controls (CON) group. All our controls were strictly having clear vessels by angiography. Exclusion criteria for this group included among others diseases such as cancer, autoimmune disease, or any other disorders likely to interact with variables under investigation. This study was performed in accordance with the regulations laid down by the Hospital Ethics Committee and all participants signed an informed consent.

**Table 1:** Demographic data and CAD risk factors distribution among anigiographed controls (CON) and CAD patients

		**CAD**	**CON**
***Demographic data***			
No of patients		1054	762
Sex	M	642	464
	F	412	298
Mean age ± SD	M	52 ± 7.5 y	53 ± 6.1 y
	F	57 ± 7 y	55 ± 3.2 y

***CAD risk factors***			
Family history (FH) of CAD		290 (27.5%)	162 (21.2%)
Obesity		723 (68.6%)	345 (45.3%)
Hypertriglyceridemia		616 (58.4%)	283 (37.1%)
Hypercholesterolemia		685 (65%)	307 (40.3%)
Diabetes Mellitus (DM)		592 (56.2%)	301 (39.5%)
Smokers		424 (40.2%)	114 (15%)
Hypertension		965 (91.5%)	497 (65.2%)
Myocardial infarction (MI)		103 (9.7%)	55 (7.2%)

DM, Diabetes Mellitus; FH, family history of CAD; MI, Myocardial infarction on admission; Hypertriglyceridemia (> 1.8 mmol/L); Hypercholesterolemia (> 5.2 mmol/L) and Obesity (Body Mass Index ≥ 30).

### Sample collection, DNA extraction and determination of CAD risk factors

Five ml of peripheral blood were collected in EDTA tubes from all participating individuals after obtaining their written consent. DNA extraction was performed using the PURGENE DNA isolation kit from Gentra Systems (Minneapolis, USA), and stored in aliquots at -20°C until required. Total serum cholesterol and triglyceride levels were measured as routine in the main hospital pathology laboratory on Konelab 20XTi clinical chemistry analyzer utilizing specific kits according to the manufacturer's protocol. Calibration of the analyzer was performed daily using internal controls prior to each session as per instruction of the manufacturer. Triglyceride levels > 1.8 mmol/L and cholesterol levels > 5.2 mmol/L were considered elevated. Diabetic patients either had a known history of diabetes mellitus or were diagnosed as such according to the American Diabetes Association criteria [15]. Individuals with body mass indices (BMI) ≥ 30 were considered obese in accordance with the Center for Disease Control and Prevention (Atlanta, GA, USA). Diagnosis of myocardial infarction was based on the consensus specified by the European Society of Cardiology and the American College of Cardiology [16]. Information about all other risk factors was procured either through patient interviews or by referring to their medical records.

### Detection of GSTT1 and GSTM1 deletions

Determination of the GSTT1 and GSTM1 deletions was carried out by multiplex polymerase chain reaction (PCR) amplification using the following primers: GSTT1 forward primer 5'- TT CCT TAC TGG TCC TCA CAT CTC -3'; GSTT1 reverse primer 5'- TCA CCG GAT CAT GGC CAG CA -3'; GST M1 forward primer: 5'- GAA CTC CCT GAA AAG CTA AAG C -3' and GSTM1 reverse primer 5'- GTT GGG CTC AAA TAT ACG GTG G -3'. Each 25 μl PCR reaction contained 2.5 μl of 10X reaction buffer with MgCl_2 _(Amersham Pharmacia Biotech, Piscataway, NJ, USA), 10 ρ mol of each primer, 100 ρ mol/μl each of deoxynucleoside triphosphates (deoxyadenosine triphosphate, deoxyguanosine triphosphate, deoxycytidine triphosphate and deoxythymidine triphosphate) (Perkin-Elmer Corporation, Foster City, CA, USA) in Tris HCl buffer, 1 unit of HotStar Taq DNA polymerase (Amersham Pharmacia Biotech, Piscataway, NJ, USA) and 100 ng genomic DNA template at annealing temperature of 58°C for 40 cycles. The PCR products were visualized on a 2% agarose gel electrophoresed at 100V for 50 minutes. Two bands of 459 for GSTT1 and 209 bp for GSTM1 were obtained for the T^wild ^M ^wild ^genotype. The T^wild ^M ^null ^genotype showed only one band of 459 bp and T ^null ^M ^wild ^a band of 209 bp. For T ^null ^M ^null ^genotype (homozygous absence or deletion genotype is designated as null genotype) no bands were obtained, necessitating the use of β-globin as internal positive control, in order to distinguish the null genotype from aborted PCR reactions.

### Statistical analysis

Genotype frequencies in both groups were compared by Chi-Square test. Multivariable logistic regression was used to study the effect of the GSTT1 and GSTM1 genotypes on CAD, incorporating other variables (CAD risk factors) into the model. A two-tailed *p *value <.05 was considered statistically significant and odds ratio with 95% confidence intervals are reported. All analyses were performed using SPSS v.10 (SPSS Inc., Chicago, USA) statistical analysis software.

## Results

Univariate analysis showed that the following variables were associated with the manifestation of CAD: GSTT1 genotype (*p *< .001), GSTM genotype (*p *< .001), haplotype GSTT1 plus GSTM1 (*p *< .001), smoking, age, elevated cholesterol and triglycerides, diabetes mellitus (DM), family history of CAD (FH), hypertension and obesity, whereas gender (*p *= .1) and myocardial infarction (MI) on admission (*p *= .067) were not. The variables showing an association (*p *< .05) in Table 2 were then put into a stepwise multiple logistic regression, in order to study the possible combined effect of the GSTT1 plus GSTM1 haplotype with other risk factors on CAD manifestation. The variables T^null ^M^null ^genotype, T^wild ^M^null ^genotype, T^null ^M^wild ^genotype, obesity and hypertension were retained in the model were (Table 3), whereas the interaction of the haplotype T^null ^M^null ^with smoking was not. Because of this, we decided to carry out a multiple stepwise logistic regression with each genotype separately. We first entered the GSTT1 genotype (variable) with other CAD risk factors into analysis. The only variables retained in the model then were genotypes, hypertension, hypercholesterolemia, obesity and smoking (Table 4). The interaction between smoking and genotypes with other CAD risk factors was not retained. We then entered the GSTM1 genotype (variable) into a stepwise multiple logistic regression with CAD risk factors, resulting in the retention of the genotype, hypertension, elevated cholesterol, obesity, smoking and genotype-smoking interaction (Table 5).

**Table 2:** Crude odds ratio for all CAD risk factors among CAD and CON groups

**Risk factor**	***Status***	**CAD**	**CON**	**Odds ratio**	**[95% C.I]**	***p *value**
Genotype (haplotype)	T^wild ^M ^wild^	311	627	Reference	-	-
T1 and M1 genes	T^wild ^M^null^	280	69	8.17	6.01 – 11.1	< .001
	T^null ^M^wild^	88	18	9.84	5.68 – 17.2	< .001
	T^null ^M^null^	375	48	15.7	11.2 – 22.2	< .001
Genotype (T1 gene)	T ^wild^	591	696	Reference	-	-
	T ^null^	463	66	8.26	6.19 – 11.05	< .001
Genotype (M1 gene)	M ^wild^	399	645	Reference	-	-
	M ^null^	655	117	9.05	7.12 – 11.5	< .001
Smoking	No	630	648	Reference	-	-
	Yes	424	114	3.83	3.01 – 4.87	< .001
Age	< 40	172	165	Reference	-	-
	**≥ **g40	882	597	1.42	1.11 – 1.81	.004
Hypercholesterolemia	No	369	455	Reference	-	-
	Yes	685	307	2.71	2.27 – 3.34	< .001
Hypertriglyceridemia	No	438	479	Reference	-	-
	Yes	616	283	2.38	1.97 – 2.89	< .001
DM	No	462	461	Reference	-	-
	Yes	592	301	1.96	1.62 – 2.37	< .001
FH	No	764	600	Reference	-	-
	Yes	290	162	1.40	1.13 – 1.75	.002
Gender	F	412	298	Reference	-	-
	M	642	464	1.0	0.82 – 1.22	.1
Hypertension	No	89	265	Reference	-	-
	Yes	965	497	5.77	4.41 – 7.59	< .001
Obesity	No	331	417	Reference	-	-
	Yes	723	345	2.64	2.17 – 3.22	< .001
MI	No	951	707	Reference	-	-
	Yes	103	55	1.39	0.98 – 1.99	.067

**Table 3:** Results of stepwise multiple logistic regression analysis for the GSTM1 and GSTT1 haplotype: final significant variables in the model

**Risk Factor (variables)**	**Odds ratio**	**[95% C.I]**	***p *value**
T^wild ^M^null^	8.90	6.36 – 12.5	< .001
T^null ^M^wild^	14.1	7.70 – 25.6	< .001
T^null ^M^null^	17.7	12.4 – 25.3	< .001
Obesity	2.32	1.75 – 3.08	< .001
Hypertension	2.43	1.68 – 3.53	< .001

**Table 4:** Results of stepwise multiple logistic regression analysis for the GSTT1 genotype (variable): final significant variables in the model

**Risk Factor (variables)**	**Odds ratio**	**[95% C.I]**	***p*value**
GSTT1 Genotype (T^wild ^vs. T^null^)	6.24	4.49 – 8.67	< .001
Hypertension	3.11	2.19 – 4.44	< .001
Hypercholesterolemia	1.57	1.22 – 2.02	< .001
Obesity	2.10	1.61 – 2.73	< .001
Smoking	1.96	1.45 – 2.64	< .001

GSTT1 genotype × smoking interaction was entered jointly into the multiple logistic regression analysis. Other factors were entered disjointedly. After analysis, the genotype × smoking interaction was not retained in the model and therefore is not shown in the above table.

**Table 5:** Results of stepwise multiple logistic regression analysis for the GSTM1 genotype (variable): final significant variables in the model

**Risk Factor (variables)**	**Odds ratio**	**[95% C.I]**	***p*value**
GSTM1 Genotype (M^wild ^vs. M^null^)	8.90	6.32 – 12.5	< .001
Hypertension	2.49	1.74 – 3.57	< .001
Hypercholesterolemia	1.39	1.10 – 1.81	.015
Obesity	2.10	1.59 – 2.75	< .001
Smoking	2.82	1.87 – 4.28	< .001
Genotype (M^wild ^vs. M^null^) × smoking	0.469	0.26 – 0.85	.012

GSTM1 genotype × smoking interaction was entered jointly into the multiple logistic regression analysis. Other factors were entered disjointedly. After analysis, the genotype × smoking interaction was retained in the model and therefore is shown in the above table.

The results for binary logistic regression model for smoking and GSTM1 genotype (variable) alone (with no CAD risk factors involved) were positive. Similar observations were made for the GSTT1 genotype and smoking, indicative of an interaction between these two genotypes and smoking. This significance was however lost, when we entered the GSTT1 genotype × smoking jointly with other CAD risk factors (Table 4), but retained when we entered the GSTM1 genotype × smoking jointly with other CAD risk factors (Table 5).

Since we found significant interaction for smoking with the genotype, we proceeded to stratify the data based on smoking status. Although the GSTT1 genotype-smoking interaction was not significant in the multivariate analysis (Table 4), we included it nonetheless for consistency, especially since smoking and this interaction had shown significant association with CAD. In the subgroup analysis, we found a significant difference between the CAD and CON groups in the distribution of the T^null ^among smokers [odds ratio = 4.4; 95% CI (2.7–6.9); *p *< .001] and non-smokers [odds ratio = 8.1; 95% CI (5.3–12.6); *p *< .001]. Similarly, the prevalence of the M^null ^among smokers [odds ratio = 5; 95% CI (3.2–7.9); *p *< .001] and non-smokers [odds ratio = 8.7; 95% CI (6.4–11.9); *p *< .001] also exhibited significant difference between the groups (Table 6).

**Table 6:** Distribution of genotypes among smokers and non-smokers

**Risk factor**	***Status***	**CAD**	**CON**	**Odds ratio**	**[95% C.I]**	***p *value**
Smokers	T^wild^	137	77	Reference	-	-
	T^null^	287	37	4.4	2.7 – 6.9	< .001
Non-smokers	T^wild^	456	619	Reference	-	-
	T^null^	174	29	8.1	5.3 – 12.6	< .001

Smokers	M^wild^	84	63	Reference	-	-
	M^null^	340	51	5	3.2 – 7.9	< .001
Non-smokers	M^wild^	317	582	Reference	-	-
	M^null^	313	66	8.7	6.4 – 11.9	< .001

Wild, deletion is not present; Null, deletion is present.

## Discussion

This study provided us with an excellent opportunity to explore the effects of gene polymorphisms, gene-gene and genotype – cigarette smoking interactions, as well as its possible effect on the development of CAD. To our knowledge, this is the first study to evaluate the prevalence of the GSTT1 and GSTM1 gene polymorphisms in an Arab population. Among our angiographed controls, the GST M ^null ^frequency was 0.153, which is lower than the values of 0.527, 0.550, 0.513, 0.497, 0.240, 0.299 observed among Australians, Brazilians, Canadians, Chinese, Indians and Black Americans [17] respectively. Also, the GST T^null ^frequency in this group was 0.09, which is lower than the values of 0.184, 0.185, 0.172, 0.467, 0.182 and 0.231 observed among Australians, Brazilians, Canadians, Chinese, Indians and Black Americans [5,17]. However, we were unable to determine whether these polymorphisms were in Hardy-Weinberg equilibrium, because heterozygous individuals could not be distinguished from homozygous wild type. Besides, it would be difficult for us to test for the equilibrium, since random mating in the Saudi population is not satisfied because of the high consanguinity rate (> 65%) [18]. We can not offer a good explanation, other than ethnicity-based variations, as to why the prevalence rates of these two genotypes are lower in our population, especially since our study involved a reasonably large number of controls.

The univariate analysis showed that the genotypes T^null ^and M^null^, the haplotype T^null ^plus M^null^, as well as smoking, age, elevated cholesterol and triglycerides, diabetes, family history of CAD, hypertension and obesity were associated with CAD, whereas gender and myocardial infarction were not. Surprisingly, myocardial infarction on admission was not associated with CAD (p = .067), possibly due to the small number of CAD patients with this disorder (9.8%). The association of the GST T^null ^genotype, M^null ^genotype, T^null ^plus M^null ^haplotype with CAD observed in our study was not in concordance with the finding of Girisha et al [5], who found no association of the GST M^null ^genotype with CAD in North Indian population. Also, Wilson et al [10] reported that the GST M^null ^genotype was not significantly associated with the incidence of CAD among South Asians. On the other hand, our finding of an association of the GST T^null ^genotype with CAD was in agreement with the report by Tamer et al [6], but not with other previous studies [5,8,10], which did not find an association.

Our multivariate analysis indicated that genotypes T^null^, M^null ^and haplotype T^null ^plus M^null ^are strongly associated with CAD in the presence of some CAD risk factors. However, the interaction of smoking with genotype was only significant in case of the GSTM1 genotype, and this may be because GSTM1 products are more directly involved than GSTT1 in the detoxification of the main carcinogen found in the tobacco smoke. However, when we studied the prevalence of the T^null ^and M^null ^genotypes among smokers and non-smokers with and without CAD, we found a significant association with CAD among smokers and non-smokers, with a stronger relation among non-smokers. Thus, our results indicate significant relevance of these two genotypes for the development of CAD, independent of the smoking status. This finding is in agreement with the studies by Wilson et al [10] and Wang et al [19] and discordant with those of Tamer et al [6] and Masetti et al [8], thus pointing to ethnic variability.

Our findings of significant relevance of these two genotypes for the development of CAD, independent of the smoking status might not be surprising. GST enzymes, beside their role in detoxifying polycyclic aromatic hydrocarbons found in tobacco smoke, involve in detoxification of many chemical carcinogens, environmental pollutants and anti-tumor agents. In addition, GST enzymes inactivate endogenous unsaturated aldehydes, quinines, epoxides and hydroperoxides formed as secondary metabolites during the oxidative stress [20]. GST enzymes also play a key role in protecting blood vessels against endogenous oxidants [20]. Therefore, absence of or reduced GST enzyme activities may lead to malfunction in the inactivation of these metabolites, generated during the oxidative stress process, and this may contribute to the development of atherosclerotic CAD. This indicates that the presence of these deletion alleles (M^null ^and T^null^) may compromise one's capabilities for detoxification of different endogenous and exogenous oxidants, other than those found in cigarette smoke, and ultimately put one at higher risk of developing CAD, even if one is not a smoker.

## Conclusion

We can conclude the following from this study: i) smoking, T^null ^and M^null ^genotypes are independent risk factors for CAD and ii) there is evidence of genotype-smoking interaction, which however does not increase the chances of developing CAD.

## Abbreviations

GST Glutathione S-transferase

CAD Coronary artery disease

CI Confidence interval

CON Control group

DM Diabetes mellitus

FH Family history of CAD

MI Myocardial infarction

PCR Polymerase chain reaction

## Competing interests

The author(s) declare that they have no competing interests.

## Authors' contributions

KKA was in charge of designing and analysis of data, OMA performed the technical aspects of the study, PCR and genotyping, GHM performed the statistical analysis and ND was responsible for recruiting patients and overall supervision of the study.

## Pre-publication history

The pre-publication history for this paper can be accessed here:


